# The Biosafety Research Road Map: The Search for Evidence to Support Practices in the Laboratory—Mpox/Monkeypox Virus

**DOI:** 10.1089/apb.2022.0045

**Published:** 2023-09-12

**Authors:** Stuart D. Blacksell, Sandhya Dhawan, Marina Kusumoto, Kim Khanh Le, Kathrin Summermatter, Joseph O'Keefe, Joseph Kozlovac, Salama Suhail Al Muhairi, Indrawati Sendow, Christina M. Scheel, Anthony Ahumibe, Zibusiso M. Masuku, Allan M. Bennett, Kazunobu Kojima, David R. Harper, Keith Hamilton

**Affiliations:** ^1^ Mahidol-Oxford Tropical Research Medicine Unit, Faculty of Tropical Medicine, Mahidol University, Bangkok, Thailand.; ^2^ Centre for Tropical Medicine and Global Health, Nuffield Department of Medicine, Nuffield Department of Medicine Research Building, University of Oxford, Oxford, United Kingdom.; ^3^ Institute for Infectious Diseases, University of Bern, Bern, Switzerland.; ^4^ Ministry for Primary Industries, Wellington, New Zealand.; ^5^ United States Department of Agriculture, Agricultural Research Service, Beltsville, Maryland, USA.; ^6^ Abu Dhabi Agriculture and Food Safety Authority, Abu Dhabi, United Arab Emirates.; ^7^ Indonesian Research Center for Veterinary Science, National Research and Innovation Agency, Bogor, Indonesia.; ^8^ WHO Collaborating Center for Biosafety and Biosecurity, Office of the Associate Director for Laboratory Science, Center for Global Health, Centers for Disease Control and Prevention, Atlanta, Georgia, USA.; ^9^ Nigeria Centre for Disease Control and Prevention, Abuja, Nigeria.; ^10^ National Institute for Communicable Diseases of the National Health Laboratory Services, Johannesburg, South Africa.; ^11^ U.K. Health Security Agency, London, United Kingdom.; ^12^ Department of Epidemic and Pandemic Preparedness and Prevention, World Health Organization (WHO), Geneva, Switzerland.; ^13^ The Royal Institute of International Affairs, London, United Kingdom.; ^14^ World Organisation for Animal Health (OIE), Paris, France.

**Keywords:** mpox virus, mpox, pathogen characteristics, biosafety evidence, biosafety knowledge gaps

## Abstract

**Introduction:**

The virus formerly known as monkeypox virus, now called mpoxv, belongs to the Orthopoxvirus genus and can cause mpox disease through both animal-to-human and human-to-human transmission. The unexpected spread of mpoxv among humans has prompted the World Health Organization (WHO) to declare a Public Health Emergency of International Concern (PHEIC).

**Methods:**

We conducted a literature search to identify the gaps in biosafety, focusing on five main areas: how the infection enters the body and spreads, how much of the virus is needed to cause infection, infections acquired in the lab, accidental release of the virus, and strategies for disinfecting and decontaminating the area.

**Discussion:**

The recent PHEIC has shown that there are gaps in our knowledge of biosafety when it comes to mpoxv. We need to better understand where this virus might be found, how much of it can spread from person-to-person, what are the effective control measures, and how to safely clean up contaminated areas. By gathering more biosafety evidence, we can make better decisions to protect people from this zoonotic agent, which has recently become more common in the human population.

## Introduction

The World Organisation for Animal Health, The World Health Organization (WHO), and Chatham House are collaborating to improve the sustainable implementation of laboratory biological risk management, particularly in low-resource settings. The Biosafety Research Roadmap project aims at supporting the application of laboratory biological risk management and improving laboratory sustainability by providing an evidence base for biosafety measures (including engineering controls) and evidence-based biosafety options for low-resource settings.

This will inform strategic decisions on global health security and investments in laboratory systems. This work involves assessing the current evidence base required for implementing laboratory biological risk management, aiming at providing better access to evidence, identifying research and capability gaps that need to be addressed, and providing recommendations on how an evidence-based approach can support biosafety.

This manuscript presents the general characteristics of mpoxv, previously known as Monkeypox virus,^1^ the current biosafety evidence, and available information regarding laboratory-acquired infections (LAIs) and laboratory releases.

## Methods

A 15-member technical working group (TWG) was formed to develop a Biosafety Research Roadmap to support the application of laboratory biological risk management and improve laboratory sustainability by providing an evidence base for biosafety measures. The TWG conducted a gap analysis for a selected list of priority pathogens on the procedures related to diagnostic testing and associated research for those pathogens, including but not limited to sample processing, testing, animal models, tissue processing, necropsy, culture, storage, waste disposal, and decontamination.

To achieve this, databases, websites, publications, reviews, articles, and reference libraries were screened for relevant data. The main research domains used to perform the literature searches were the ABSA database, Belgian Biosafety Server, CDC reports, WHO reports, PubMed, and internet searches for terms related to biosafety matters, including, for example, inactivation, decontamination, LAIs, laboratory releases, and modes of transmission. Blacksell et al.^2^ provided a detailed description of the materials and methods and an introduction to why the gap analysis was performed.

### General Characteristics

Mpoxv is a viral zoonosis belonging to the genus *Orthopoxvirus* and family *Poxviridae* in risk group 3.^3^ Mpoxv is a 200 to 250 nm brick-shaped, enveloped virus with characteristic surface tubules and a dumbbell-shaped core component (adapted from reference 1). Mpoxv is divided into three clades: Clade I (formerly the Central African/Congo Basin Clade), Clade IIa, and Clade IIb (both formerly West African Clade).^4^

Studies have shown that the Clade I mpoxv strain is genetically distinct from and more virulent than Clades IIa and IIb as distinguished by genetic sequencing and clinical signs and symptoms.^5–7^ Signs and symptoms of mpox infection may include flu-like symptoms (fever, chills, fatigue, headache, and muscle ache), swollen lymph nodes, and a rash. Symptomology and the severity of symptoms vary among infected individuals.

The rash typically starts as flat red spots and progresses for 2–4 weeks through stages of forming hard red bumps, fluid-filled blisters, and blisters filled with pus, terminating with scabs that fall off.^8^ Mpoxv usually circulates in Central and Western Africa;^9^ however, outbreaks were reported in 2003 in the United States and numerous non-mpox endemic countries in 2022^10,11^ across five WHO regions.^12^ While a telltale symptom of infection by most viruses in the *Orthopox* genus is the formation of vesicular-pustular lesions, a clear distinction can be made between variola virus, vaccinia virus, cowpox virus, and mpoxv by genetic analysis.^13–15^

#### Treatment and prophylaxis

At the time of this writing, no approved treatment is specific for mpoxv infections; treatment is supportive and based on alleviating symptoms.^3,16,17^ Tecovirimat is FDA-approved for treating smallpox but not other poxvirus diseases. It is available for clinical use under an expanded access Investigational New Drug (EA-IND) protocol and has demonstrated efficacy in treating the illness and improving disease outcomes.^18,19^

The European Medicines Agency approved it for mpox treatment in 2022, but it has yet to be widely available.^17^ A Modified Vaccinia Ankara vaccine, JYNNEOS, is available under emergency authorization. It is a two-dose (also known as Imvamune or Imvanex) used to protect against mpox and smallpox infection.^20,21^ While the ACAM2000 vaccine is approved for immunization against smallpox and was made available for use against mpox in 2022 in the United States under an EA-IND protocol,^22^ it is not as widely used as there are more known side effects and contraindications than the JYNNEOS vaccine.^23^

#### Diagnostics

Tests that provide definitive and rapid diagnosis of mpox infection include nucleic acid amplification tests (i.e., polymerase chain reaction [PCR]) and real-time quantitative PCR (RT-qPCR) alone or in conjunction with viral genome sequencing.^16,24–26^ RT-qPCR and sequencing may not be feasible in resource-constrained settings due to equipment, reagent costs, and infrastructure capability (i.e., reliable electrical supply).

Serological tests, including virus neutralization tests, hemagglutination-inhibition, immunofluorescence, enzyme-linked immunosorbent assays, and Western blot, may not provide a definitive diagnosis of mpox infection if there is cross-reactivity between reagents used to identify mpox viral antigens and viral antigens from other viruses in the *Orthopoxvirus* family.^13,16,27,28^ Mpox virus can also be detected in clinical samples by electron microscopy and procedures involving viral isolation.^26,28^

## Biosafety Evidence

### Modes of Transmission

Mpox is a zoonotic disease maintained in small animals. Reservoir species in central and west Africa include sun squirrels, giant pouched rats, African dormice, and other rodents.^16^ Historically, mpoxv infections in humans have been attributed to close contact with virus-carrying animals, and human-to-human transmission was considered rare.^16,27,29^

In 2003, mpox infections were reported in the United States when patients were exposed to domesticated prairie dogs (*Cynomys* spp.) that were housed with various exotic African rodents (*Funiscuirus* spp., *Heliosciurus* spp., *Cricetomys* spp., *Atherurus* spp., *Graphiurus* spp. and *Hybomys* spp.) shipped from Ghana to the United States as part of the pet trade.^30,31^ In this instance, another mode of transmission other than “close contact” occurred as the animals were in the same room but separately housed.

The multi-country outbreak that originated in May 2022 demonstrates that mpox is transmissible between humans most prevalently via direct, skin-to-skin contact, contact with skin lesions or scabs, or by indirect contact with contaminated materials such as bedding, clothing, porous furniture, eating utensils, or from inhalation of respiratory droplets during prolonged periods of face-to-face contact.^32^

Research has indicated the presence of mpoxv on all surfaces touched by infected patients with high viral loads. Surface isolates tested demonstrated at least 10^6^ copies of the virus per sample, indicating that contaminated surfaces with higher viral loads may be potentially infectious.^33^ It has also been recovered from the air during linen changes and the doffing of personal protective equipment (PPE).^34^

### Infectious Dose

There are no definitive data on the minimum infectious dose required to cause mpox infections in humans. Several studies in animal models have demonstrated infectious doses experimentally for reservoirs of rabbits, rats, pigs, non-human primates, and squirrels^35–37^

However, many studies related to vaccine challenge studies rather than determining the minimum infectious dose. A review of animal models cites the LD_50_ of mpoxv in Prairie dogs to be 5.9 × 10^3^ pfu when administered via the intranasal route.^38^ The aerosol LD_50_ for non-human primates with Clade 1 has been reported as 7.8 × 10^4^ pfu.^39^

### Laboratory-Acquired Infections

There have not been any documented cases of mpoxv laboratory infections reported in the scientific literature. However, mpoxv infections involving sharps injuries have been reported in health care settings.^40–42^ There have been multiple instances of laboratory-acquired exposures to other orthopoxviruses and subsequent infections, predominantly with the vaccinia virus.^43^

### Disinfection and Decontamination

#### Chemical

Vaccinia viruses could be inactivated by at least 4 log10 in suspension tests and on artificially contaminated surfaces by 70% ethanol (1 min) or 0.2% peracetic acid (10 min), as demonstrated primarily with various organic loads.^44^ In suspension tests, hydrogen peroxide (14.4%) and iodine (0.04–1%) were effective, whereas sodium hypochlorite (0.25–2.5%; 1 min), 2% glutaraldehyde (10 min), and 0.55% orthophthalaldehyde (5 min) were effective on artificially contaminated surfaces.^44^

Vaccinia virus was demonstrated to be inactivated by Virkon^®^. Virkon used in the study contained 50% w/w potassium peroxomonosulfate, 5% sulfamic acid, and 15% sodium alkylbenzene sulfonate.^45^ After 3 min of exposure, copper surfaces (99.9%), such as those utilized in laboratories, lowered vaccinia virus and mpoxv titers by 4 log10, whereas stainless steel was substantially less efficient.^46^ Inactivation of vaccinia virus occurs in 2–3 h at 60°C or within minutes following exposure to 20 nM caprylate at 22°C; however, MPXV is more resistant than vaccinia to solvent-detergent treatment.^47^

The United Kingdom Health Security Agency has published evidence of the effectiveness of various commercial extraction buffers and transport mediums and the efficacy of inactivating mpoxv in clinical specimens (Table 1). The following were effective at mpoxv inactivation: NeuMoDx Vantage Viral Lysis Buffer^48^ [50.5% guanidine hydrochloride; 0.8% sodium tetraborate decahydrate; 0.3% Tris(2-carboxyethyl)phosphine hydrochloride], Zymo Research DNA/RNA Shield Buffer^49^ (SafeCollect Swab Tube), Qiagen Buffer ATL^50^ [Sodium dodecyl sulfate (≥1 < 10% w/w)], Roche Cobas PCR Media^51^ [≤40% (w/w) guanidine hydrochloride], Longhorn Vaccines & Diagnostics PrimeStore Molecular Transport Medium^52^ (20–30% guanidine thiocyanate; 19–25% ethanol; <0.7% N-Lauroylsarcosine Na+), Qiagen Buffer AVL^53^ (50–70% w/w guanidinium thiocyanate), 70% Ethanol,^54^ Thermo Scientific InhibiSURE Viral Inactivation Medium,^55^ Severn Biotech Ltd L6 Buffer,^56^ BioServ MagBead Viral RNA Lysis Buffer^57^ (1–2% w/v dl-dithiothreitol; 20–30% w/v guanidine thiocyanate), Hologic Panther Fusion Specimen Lysis Tubes,^58^ and E&O Laboratories Ltd Molecular Sample Solution^59^ (40–50% guanidine thiocyanate; 0.5–1% Tergitol 15-S-9 surfactant).

**Table 1.** Detailed pathogen biosafety evidence for mpoxv

***Overview of the evidence and potential gaps in biosafety***				
***Method***	***Details***	***Evidence (direct quote where available)***	***Reference***	***Evidence gap? (yes/no)***
Route of inoculation	Inhalation	“Monkeypox is transmitted to humans through close contact with an infected person or animal, or with material contaminated with the virus.”	27	No
	Cutaneous	“In this study monkeypox virus was successfully isolated from three different samples, each with a total of at least 10^6^ virus copies. Thus, contaminated surfaces with such viral loads or higher, could potentially be infectious and it cannot be ruled out that their contact with especially damaged skin or mucous membranes, could result in transmission.”	33	
		“…have been detected in males between 18–50 years, and primarily among men who have sex with men (MSM). Particular sexual practices have facilitated the transmission of MPX among MSM groups with multiple partners.”	69	
		“contact with clothing or linens (such as bedding or towels) used by an infected person, direct contact with monkeypox skin lesions or scabs, large droplet respiratory spread from prolonged close contact with an individual with a monkeypox rash.”	32	
Infectious dose	Human to human Animal to human	No information.		Yes
Laboratory-acquired infections		No reports of occupational-acquired infection of laboratory staff. Three sharps-related occupational infection reports by health care workers.	40–42	Yes
Chemical inactivation	Sodium hypochlorite, chloroxylenol-based household disinfectants, glutaraldehyde, formaldehyde, and paraformaldehyde	“Vaccinia viruses could be inactivated by at least 4 log_10_ in suspension tests and on artificially contaminated surfaces by 70% ethanol (≤1 min) or 0.2% peracetic acid (≤10 min), mostly shown with different types of organic load. Hydrogen peroxide (14.4%) and iodine (0.04–1%) were effective in suspension tests, sodium hypochlorite (0.25–2.5%; 1 min), 2% glutaraldehyde (10 min) and 0.55% orthophthalaldehyde (5 min) were effective on artificially contaminated surfaces.”	44	Partial—No distinction between mpoxv and Orthopoxviruses in some cases
	Various disinfectants	“Monkeypox belongs to a group of viruses that is more susceptible to disinfectants than other types of viruses. While there are no disinfectants registered for use against monkeypox, all products with EVP claims have been tested against viruses that are more difficult to kill than monkeypox.”	65	
	Copper	“The vaccinia virus strain Elstree and the virulent monkeypox virus strain Copenhagen were both tested on surfaces with 99.9% copper and stainless steel at room temperature. The initial viral titre of both viruses (approximately 10^6^ pfu) was reduced by ≥4 log_10_ within 3 min on copper whereas the decline was less than 2 log_10_ on stainless steel within 5 min and remained small after 20 min.”	46	
	Chloroxylenol-based household disinfectants	“At least one chloroxylenol-based household disinfectant is available, which inactivates vaccinia virus on contact.”	45	
	NeuMoDx vantage viral lysis buffer (50.5%) guanidine hydrochloride 0.8% sodium tetraborate decahydrate 0.3% Tris(2-carboxyethyl)phosphine hydrochloride)	“Treatment with NeuMoDx Vantage Viral Lysis Buffer (1 volume product to 1 volume sample) for 10 minutes reduced monkeypox virus titre to below the limit of detection for the test. This equates to ≥4.7 log10 reduction in virus titre, or a reduction of 99.998%.”	48	
	Cepheid Xpert CT/NG Swab Transport Reagent (ammonium chloride) (5–8%), potassium carbonate (0.5–1.5%)	“Treatment with Xpert CT/NG Swab Transport Reagent for 30 minutes failed to inactivate monkeypox virus in this test. **This product should not be relied upon to inactivate monkeypox virus.”**	60	
	Zymo research DNA/RNA Shield buffer (SafeCollect Swab Tube)	“Treatment with DNA/RNA Shield for 10 minutes or more reduced monkeypox virus titre by ≥2.6 log10 FFU/mL. This equates to a reduction of ≥99.77%. Although treatment with this product reduced the level of infectious virus to below the limit of detection of the assay, considerable product cytotoxicity remained following sample purification that reduced the sensitivity of the test.”	49	
	Qiagen buffer ATL (sodium dodecyl sulphate (≥1 < 10% w/w))	“Treatment with ATL Buffer (1 volume product to 1 volume sample) for at least 15 minutes reduced monkeypox virus titre by 5.3–5.4 log10, or a reduction of 99.9995%. Low levels of residual virus (13–18 FFU/mL) were detected in 5 out of 6 treated sample replicates.”	50	
	NeuMoDx viral lysis buffer (Guanidine hydrochloride, 33.5%)	“Treatment with NeuMoDx Viral Lysis Buffer (1 volume product to 1 volume sample) for 10 minutes resulted in a 1.1 log10 reduction in monkeypox virus titre in these tests. This is a modest reduction compared to the inactivation effectiveness of other molecular lysis buffers, and a considerable level of infectious virus remaining following treatment with this product. **This product should not be relied upon to completely inactivate monkeypox virus.**”	61	
	Roche Cobas PCR Media (≤40% (w/w) guanidine hydrochloride)	“Treatment with Cobas PCR media for 60 minutes or more reduced monkeypox virus titre to below the limit of detection of the titration assay. This equates to a ≥ 3.9 log10 reduction in virus titre, or a reduction of ≥99.988%. Titres were also reduced by 2.6 log10 reduction, or 99.738%, following a shorter 30- minute treatment although virus was readily detectable after this treatment time.”	51	
	Longhorn vaccines and diagnostics PrimeStore molecular transport medium (20–30% guanidine thiocyanate 19–25% ethanol <0.7% N-Lauroylsarcosine Na+)	“Treatment with PrimeStore Molecular Transport Medium for 2 minutes or more reduced monkeypox virus titre to below the limit of detection of the titration assay. This equates to a ≥ 4.5 log10 reduction in virus titre, or a reduction of ≥99.997%. A previous version of this report showed that treatment with PrimeStore Molecular Transport Medium for 30 minutes or more reduced monkeypox virus titre by ≥4.4 log10, or ≥99.996%.”	52	
	Qiagen Buffer AVL (50–70% w/w guanidinium thiocyanate)	Treatment with Buffer AVL for 10 minutes reduced virus titer to below the limit of detection of the titration assay. This equates to a ≥ 4.0 log10 reduction in virus titer, or a reduction of ≥99.991%.	53	
	70% Ethanol	“Treatment with 70% Ethanol for 10 minutes reduced virus titre to below the limit of detection of the titration assay. This equates to a ≥ 4.5 log10 reduction in virus titre, or a reduction of ≥99.997%.”	54	
	Thermo Scientific InhibiSURE viral inactivation medium	“Treatment with InhibiSURE Viral Inactivation Medium for 10 minutes or more reduced monkeypox virus titre to below the limit of detection of the titration assay. This equates to a ≥ 4.4 log10 reduction in virus titre, or a reduction of ≥99.996%.”	55	
	Severn Biotech Ltd L6 buffer	“Treatment with L6 Buffer for 10 minutes reduced monkeypox virus titre to below the limit of detection of the titration assay. This equates to a ≥ 4.1 log10 reduction in virus titre, or a reduction of ≥99.992%.”	56	
	BioServ MagBead viral RNA lysis buffer (1–2% w/v dl-dithiothreitol; 20–30% w/v guanidine thiocyanate)	“Treatment with 2x MagBead Viral Lysis Buffer (1 volume product to 1 volume sample) for 10 minutes reduced monkeypox virus titre to below the limit of detection of the titration assay. This equates to a ≥ 4.1 log10 reduction in virus titre, or a reduction of ≥99.992%.”	57	
	Hologic Panther Fusion Specimen Lysis Tubes	“Treatment with Hologic STM (1.42 volumes product to 1 volume sample) for 10 minutes reduced monkeypox virus titre to below the limit of detection of the titration assay. This equates to a ≥ 5.1 log10 reduction in virus titre, or a reduction of ≥99.999%. This study evaluated inactivation of monkeypox virus by STM under conditions compatible with the instructions for use of Hologic Panther Fusion Specimen Lysis Tubes, e.g., 500 μl liquid specimen into a Panther Fusion Specimen Lysis Tube. Hologic STM is also a component of the Aptima Multitest Swab Specimen Collection Kit (PRD- 03546), which requires a swab to be collected directly into a tube containing 2.9 ml of STM. Based on data presented in this study, Aptima transport tubes are therefore also likely to inactivate monkeypox virus effectively.”	58	
	E&O Laboratories Ltd Molecular sample solution (40–50% guanidine thiocyanate 0.5–1% Tergitol 15-S-9 surfactant)	“Treatment with MSS (1 volume product to 1 volume sample) for 30 minutes reduced monkeypox virus titre to below the limit of detection of the titration assay. This equates to a ≥ 4.8 log10 reduction in virus titre, or a reduction of ≥99.998%.”	59	
	20 nM Caprylate	“Complete inactivation of the closely related vaccinia virus …. within minutes following exposure to 20 nM caprylate at 22°C; however, MPXV is more resistant than vaccinia to solvent-detergent treatment.”	47	
Thermal inactivation or autoclaving		“…in combination with standard sterilization procedures with an autoclave (which is commonly available in almost every medical facility) for inactivation of smallpox virus in patient specimens, permits the laboratory diagnosis of smallpox virus infections caused by possible bioterrorism events by qualified laboratories at the local level.”	62	Partial—No distinction between mpoxv and Orthopoxviruses
		“Physical Inactivation: Orthopoxviruses are inactivated by heat (autoclaving and incineration).”	3	
		Complete inactivation of the closely related vaccinia virus occurs in 2–3 h at 60°C.	47	
Gaseous		No information.		Yes

EVP, emerging viral pathogens.

It was noted that the Cepheid Xpert CT/NG Swab Transport Reagent^60^ (Ammonium chloride, 5–8%; Potassium carbonate, 0.5–1.5%), and the NeuMoDx Viral Lysis Buffer^61^ (Guanidine hydrochloride, 33.5%) were not effective at complete mpoxv inactivation. A list of the evidence is provided in Table 1.

#### Thermal and autoclaving

It has been suggested that *Orthopoxvirus* are inactivated by heat (autoclaving and incineration).^3^ Autoclave decontamination procedures in most medical facilities inactivate the mpox virus in patient specimens.^62^ Complete inactivation of the closely related vaccinia virus occurs in 2–3 h at 60°C^47^ (Table 1).

#### Engineering controls

Patient isolation is recommended for infection control in health care settings. Patients with suspected or confirmed mpox infection should be placed in a single-person room. Although special air handling is not required, the door should be kept closed. Any procedures likely to generate oral secretions (i.e., those involving intubation or extubation) should be performed in an airborne infection isolation room. The PPE for health care professionals includes gloves, gowns, goggles or full-length face shields, and respiratory protection (i.e., N95).^63^

Work involving diagnostic samples should be conducted in a Biosafety Level 2 (BSL-2) laboratory (preferably with inward directional airflow), and using Class II Biosafety Cabinet (BSC) or other containment devices, especially if there is a potential to generate aerosols. The PPE may include solid-front gowns with cuffed sleeves, double gloves, eye protection (safety glasses, snugly fitting goggles) or face protection (face shield), and a particulate respirator (i.e., N95 or higher).^64^

A site-specific risk assessment that informs PPE use, primary containment, and practices should, at a minimum, consider the exposure risk posed by the procedure, proficiency of the individual conducting the work, availability of containment equipment, and whether staff have been vaccinated for smallpox or mpox.^64^

Work on the open bench if a Class II BSC is not available is per a site-specific risk assessment and should include provisions for limiting the number of people in the laboratory and a combination of PPE and other containment devices (e.g., glove box, centrifuge safety cups, or sealed rotor) to create a barrier between the specimen and laboratory personnel.^64^

Work involving in vitro mpoxv culture should be performed at BSL-3.^64^ Staff working with mpoxv should be offered the vaccine as part of the occupational health program.

## Knowledge Gaps

### Animal Reservoir

It is still unclear where the mpoxv virus originates in the animal-to-human and human-to-human transmission routes. However, research has shown that the virus has been mainly found in a variety of hosts, including monkeys, rodents, squirrels, and prairie dogs, based on documented infection cases.

### Infectious Dose

The infectious dose is not clearly defined, especially the minimum dose required to cause mpox infection via animal-to-human and human-to-human transmission.^3,33^

### Disinfection and Decontamination

#### Chemical

According to the United States Environmental Protection Agency (EPA), there are no registered disinfectants for the mpox virus. However, the mpox virus is a group of viruses that are more sensitive to disinfectants than other viruses because the disinfectants can easily break the lipid envelope surrounding the virus, so the list of disinfectants published by the EPA can be used for mpox viruses based on their respective classifications (Tier 1, 2, 3).^65,66^

In addition, the Centers for Disease Control and Prevention (CDC) also recommends using disinfectants for mpoxv from the EPA.^67^ However, neither the CDC nor the EPA List Q for registered disinfectants for Emerging Viral Pathogens specifies contact time for use with mpoxv. Before disposal, the laboratory must confirm that the decontamination agent and process used have been validated.

#### Gaseous fumigation

There were no reports that describe the effectiveness of gaseous chemical fumigants commonly used (such as those containing formaldehyde, hydrogen peroxide or chlorine dioxide) in decontaminating spaces where work is being carried out involving pox viruses.

#### Laboratory-acquired infections

As mpox has circulated throughout Central and Western Africa for decades, with patients hospitalized in settings ranging from poorly to well-equipped regarding patient isolation and barrier practices, more information should be needed regarding LAIs. Recent mpox infections in Europe and the United States, where the disease is neither endemic nor typically expected on initial patient presentation, have yet to result in the issuance of LAI reports.

There were 87,377 confirmed cases in over 111 countries between May 2022 and May 2023,^68^ with only three sharps-related occupational infection reports by health care workers,^40–42^ but no reports of occupational-acquired infection of laboratory staff.

## Conclusions

Mpox is a highly contagious disease that can spread between animals and humans. Although it was previously confined to Africa and transmitted through person-to-person contact, it has recently spread to other regions, prompting the WHO to declare it a Public Health Emergency of International Concern in 2022.

The primary mode of transmission is via close contact with infected humans or animals. To combat the disease, it is imperative that we conduct further research to understand its spread, develop effective treatments and vaccines, and provide clear guidance on diagnosis and patient care, especially in areas with limited resources.
